# Rutin and Its Combination With Inulin Attenuate Gut Dysbiosis, the Inflammatory Status and Endoplasmic Reticulum Stress in Paneth Cells of Obese Mice Induced by High-Fat Diet

**DOI:** 10.3389/fmicb.2018.02651

**Published:** 2018-11-05

**Authors:** Xiulan Guo, Renyong Tang, Shiyong Yang, Yurong Lu, Jing Luo, Zhenhua Liu

**Affiliations:** ^1^School of Pharmacy and Biological Engineering, Chengdu University, Chengdu, China; ^2^School of Public Health and Health Sciences, University of Massachusetts, Amherst, MA, United States; ^3^College of Animal Science and Technology, Sichuan Agricultural University, Chengdu, China

**Keywords:** obesity, rutin, inulin, gut dysbiosis, inflammation, Paneth cells

## Abstract

Gut dysbiosis induced by high fat diet (HF) or obesity is a predisposing factor to develop diverse inflammatory diseases. Polyphenols and fibers, often eaten together, have been reported to have prebiotic actions, but their health promoting benefits still need to be further characterized and defined. This study attempted to understand how polyphenol rutin and polysaccharide inulin influence intestinal health in mouse model fed a HF (60 kcal%) diet. A total of 48 C57BL/6J mice were divided into four groups fed with a low fat (10% kcal%) control diet (LC), a high fat control diet (HC), a high-fat diet supplemented with rutin (HR), or a high-fat diet supplemented rutin and inulin (HRI) for 20 weeks. Rutin supplementation reduced the HF diet-induced increase of *Firmicutes*/*Bacteroidetes* (F/B) ratio, *Deferribacteraceae* population and plasma lipopolysaccharide (LPS) (*p* < 0.05); ameliorated inflammation as indicated by the decreased circulating inflammatory cytokines (*p* < 0.05) and the reduced expressions of intestinal inflammatory mediators (*p* < 0.05); and attenuated the endoplasmic reticulum (ER) stress in Paneth cells as indicated by the decreased expressions of the ER markers (*p* < 0.05). Compared to the rutin supplementation alone, the co-administration of rutin with inulin improved the utilization of rutin as indicated by its decreased excretion, suppressed a number of harmful bacteria including *Deferribacteraceae* and *Desulfovibrionaceae* (*p* < 0.05), and further reduced the expression of the key inflammatory cytokine TNF-α and increased the production of butyrate, despite the supplementation of inulin reversed the decrease of body weight induced by rutin supplementation due to an increased food intake. Taken together, our data demonstrated that rutin supplementation ameliorated the inflammatory status and ER stress in Paneth cells under a HF-induced obese state, and its co-administration with inulin further mitigated the inflammatory status, indicating the potential to combine polyphenol rutin and the polysaccharide inulin as a dietary strategy to ameliorate gut dysbiosis, to improve inflammatory status and thereby to reduce medical disorders associated with HF-induced obesity.

## Introduction

The prevalence of obesity has dramatically increased and has more than doubled since 1980 (Gregg and Shaw, 2017). Currently, one third of the world’s population is overweight or considered obese, and a further increase is even predicted, ∼50% by 2030 (Kelly et al., 2008; Finkelstein et al., 2012). Obesity has now been considered as a major public health issue since it is associated with an array of medical complications, including increased risk of type 2 diabetes, hypertension, cardiovascular disease, inflammatory bowel disease, and cancers (Hotamisligil, 2006). One of the responsible mechanisms for this relationship is the systemic inflammatory status associated with high fat diet or obesity. Recently, growing evidence has implicated the intestinal immune system as an important contributor to those diseases (Garidou et al., 2015; Luck et al., 2015; Monteiro-Sepulveda et al., 2015; Winer et al., 2016).

A close relationship between the gut microbiota and obesity has been demonstrated in both human and animals, with enriched *Firmicutes* and reduced *Bacteroidetes* phyla in obesity (Ley et al., 2005, 2006). Our recent research revealed that high fat (HF) diet stimulated intestinal inflammation via altering gut microbiota, and it occurred prior to the potential influence by circulating inflammatory cytokines (Guo et al., 2017), indicating, in addition to adipose tissue, HF *per se* also drives intestinal inflammation via altering gut microbiota. Data from Lee et al. (2017) reiterated the role of HF on intestinal inflammation and health by showing that HF-fed mice were more susceptible to experimental colitis, and exhibited severe colonic inflammation, accompanied by the expansion of selected pathobionts such as *Atopobium* sp. and *Proteobacteria*.

A number of studies have demonstrated that a variety of polyphenols and dietary fibers exert the properties to mitigate the microbial dysbiosis produced by high-fat diets (Delzenne et al., 2011; Karlsson et al., 2013; Etxeberria et al., 2015). For instances, the beneficial *Bacteroidetes* community is favored by wine vinegar, polyphenol-rich fruits and green tea (Lee et al., 2006; Cerezo et al., 2008; Selma et al., 2009). Quercetin or grape polyphenols attenuated *Firmicutes*/*Bacteroidetes* ratio and suppressed the growth of bacterial species associated to diet-induced obesity, resulting in lower intestinal and systemic inflammation (Etxeberria et al., 2015; Roopchand et al., 2015). Non-digestible dietary fiber exerts a significant role in promoting beneficial bacteria and enhance microbial diversity in the gut (Cani et al., 2007), which might contribute to ameliorate obesity and obesity associated disorders (Dewulf et al., 2011).

Polyphenols and dietary fiber may interact to mediate gut microbiota. Jaganath et al. (2006) have shown that combining fermentable carbohydrates accelerate the breakdown of the polyphenol rutin in an *in vitro* model of colonic fermentation (Hou et al., 2015). Moreover, a range of fermentable fibers inhibited phenolic acid production from rutin (Mansoorian et al., 2015). Also, polyphenols could influence the fermentation of the dietary fiber as polyphenols have been shown to have both anti-bacterial (Taguri et al., 2004) and prebiotic actions (Tuohy et al., 2012). Thus, there would be a reciprocal interaction between dietary polyphenols and fibers in the mediation of gut mcirobiota. The present study was to assess the potential of rutin and its co-administration with inulin to ameliorate HF-diet-induced gut microbiota dysbiosis and inflammation.

## Materials and Methods

### Animal Study

The animal protocol was approved by the Institutional Animal Care and Use Committee of Chengdu University. Forty-eight male C57BL/6J mice (4 weeks old) were randomly distributed into four groups: the low-fat control group (LC; *n* = 12), mice were fed a low-fat diet (LF) with 10% kcal from fat (D12450B; Research Diets Inc.); the high-fat control group (HC; *n* = 12), mice were fed a high-fat (HF) diet with 60% kcal from fat (D12492; Research Diets Inc.); a rutin group (HR; *n* = 12), mice fed a HF diet with rutin 6.4 mg/g diet; and a rutin + inulin group (HRI; *n* = 12), mice fed a HF diet with rutin 6.4 mg/g diet and inulin (30 mg/ml) via drinking water. The doses of rutin (about 0.01 mol/kg diet) and inulin were chosen based on previous studies (Jurgoński et al., 2012; Hoek-van den Hil et al., 2013, 2015; Hamilton et al., 2017). Rutin (≥97% purity) was supplied by Chengdu Okay Pharmaceutical Co. Ltd (Sichuan, China); and inulin (≥95% purity) was supplied by Rui Hu Biological Co. Ltd (Qinghai, China).

The mice were fed *ad libitum* and housed under a 12 h light/12 h dark cycle. Body weights and food intake were recorded every other week. During the 19th week, food and feces samples were collected, and feces excretion was recorded. After 20 weeks of feeding, all mice were euthanized with CO_2_. The blood sample was collected via heart puncture after euthanasia, and plasma was separated and stored at −80°C for later analysis of inflammatory cytokines and biochemical parameters. Following with the heart puncture, the abdomen was opened and visceral fat pads were harvested. Samples of the intestinal and colonic contents were collected, frozen immediately with liquid nitrogen, and stored at −80°C for further microbial abundance and SCFA analysis. A segment (∼2 cm) of ileum was excised and formalin-fixed for immunohistochemistry, and the intestinal mucosa was collected as described in a previous publication (Guo et al., 2017), and then immediately frozen with liquid nitrogen and stored at −80°C for later real-time PCR analysis.

### 16S rRNA Gene Sequencing and Analysis of Microbial Profile

16S rRNA gene sequencing was performed on the small intestinal contents from 32 C57BL/6J mice (*n* = 8 mice per diet group). Bacterial DNA was extracted from the intestinal contents using the QIAamp DNA Stool Mini Kit (Qiagen, Germany) according to the manufacturer’s instructions. The concentration and purity of extracted DNA were determined using the NanoDrop-2000 spectrophotometer (Thermo Fisher Scientific, Waltham, MA, United States).

The V4 region of the 16S rRNA gene was amplified used universal bacterial primers 515F (5′-GTGCCAGCMGCCGCGGTAA-3′) and 806R (5′-GGACTACHVGGGTWTCTAAT-3′) with Phusion High-Fidelity PCR Master Mix (New England Biolabs, Ipswich, MA, United States) followed by purification with Qiagen Gel Extraction Kit (Qiagen, Germany). The sequencing libraries were prepared using TruSeq DNA PCR-Free Sample Preparation Kit (Illumina, San Diego, CA, United States), sequenced on an Illumina HiSeq 2500 platform (San Diego, CA, United States), and 250 bp paired-end reads were generated. Paired-end reads were merged using FLASH (Baltimore, MD, United States), and subsequently raw tags were filtered with a specific standard to obtain the high-quality tags using QIIME version 1.7.0 (Caporaso et al., 2010). Sequences with ≥97% similarity were assigned to the same operational taxonomic units (OTUs) using Uparse software version 7.0.1001 (Edgar, 2013), and were annotated with taxonomic information based on the RDP classifier version 2.2 (Wang et al., 2007) algorithm using the Greengene database. OTU abundance data was normalized using a standard sequence number corresponding to the sample with least sequences, and subsequent analysis of alpha diversity and beta diversity were performed with QIIME. To visualize the sample differences, principal coordinate analysis (PCoA) was performed with weighted Unifrac (Lozupone and Knight, 2005). The clustering of samples was explained with the principal coordinate (PC) values. Differences in OTU abundance between groups were identified using LDA (Linear Discriminant Analysis) Effect Size (LEfSe) (Segata et al., 2011)^1^.

### Gene Expression in Small Intestine Mucosa

The mRNA expressions of inflammatory factors, 4 anti-microbial peptides, α-defensin 5, lysozyme, angiogenin 4 (ANG 4) and regenerating islet derived 3-gamma (Reg IIIγ), and ER stress markers in the intestine were measured by real-time PCR. Briefly, total RNA was extracted from the small intestine mucosa with Trizol (Invitrogen, Carlsbad, CA, United States); the concentrations of total RNA were determined spectrophotometrically (NanoDrop-2000, Thermo Fisher Scientific, Waltham, MA, United States), and cDNA was synthesized with the ExScript^TM^ RT-PCR kit (TaKaRa, Dalian, China). Real-time PCR was performed on the ViiA^TM^ 7 System (Applied Biosystems, Foster City, CA, United States) utilizing the following thermal cycling conditions: 95°C for 10 min, followed by 40 cycles of 95°C for 15 s and 60°C for 60 s. Primer sequences were chosen according to our previous studies (Liu et al., 2016; Guo et al., 2017) and listed in Supplementary Data (Table S1).

### Immunohistochemistry of Lysozyme

Immunohistochemical analysis was performed on the formalin-fixed sections of ileum. The paraffin-embedding slides were deparaffinized in xylene, followed by rehydration in ethanol. After blocking non-specific antibody binding with 5% bovine serum albumin, sections were incubated with the specific first antibody of rabbit monoclonal antibody lysozyme (ab108508; Abcam, Cambridge, United Kingdom) followed with the second antibody goat anti-rabbit IgG HRP. Immunoreactivity was detected with horseradish peroxidase-conjugated anti-goat EnVision kit (DAKO). All slides were counterstained with hematoxylin. Paneth cells were identified microscopically by their location just at the base of small intestinal crypts of Lieberkuhn (Elphick and Mahida, 2005).

### Blood Plasma Analysis

For detecting inflammatory cytokines in the blood, a ProcartaPlex Mouse High Sensitivity Panel (5 plex) from Thermo Fisher Scientific for 5 cytokines, IFN-γ, IL-2, IL-4, IL-6, and TNF-α, was selected for mice, and the assays were performed on the MAGPIXTM platforms (Luminex, Austin, TX, United States) following the manufacturer’s instruction. Plasma leptin level was determined using a leptin ELISA kit (Multisciences, Hangzhou, China) according to the manufacturer’s instructions. Triglycerides and total cholesterol contents were quantified by Beckman Coulter DXC 600 Pro (Beckman Coulter, Inc., Brea, CA, United States) using standard spectrophotometric assays. Plasma LPS level was analyzed using a mouse LPS ELISA kit (Cusabio, Wuhan, China). All standards and samples were measured in duplicate.

### Short-Chain Fatty Acid (SCFA) Analysis

Short-chain fatty acids (acetate, propionate, and butyrate) in colonic contents were determined. Colonic content samples (150 mg) were vortexed with 350 μl deionized water and 2-ethylbutyric acid (internal standard, Sigma Chemical), after which 500 μl 2.5 M sulphuric acid was added to the sample. SCFA were then extracted with diethyl ether, silylated with n-(tert-butyldimethylsilyl)-n-methyltrifluoroacetamide (Sigma Chemical), and then centrifuged (5,000 × *g*, 10 min). The supernatant was filtered through a 0.45 μm filter, and 1 μl of clear filter solution was directly injected into gas chromatograph system (Agilent Technologies) for analysis, which was performed as described by Sheveleva and Ramenskaya (2010).

### Rutin Content and Rutin Degradation Analysis

Rutin content was measured according to Araujo et al. (2013). The food samples or feces samples were mixed with the 60% methanol solution, and the suspension was extracted under ultrasonication for 50 min and subsequently centrifuged for 10 min at 5,000 × *g*. The supernatant filtered through a 0.45 μm filter was used in high performance liquid chromatography (Shimadzu Co., Kanagawa, Japan) analysis. After detecting the rutin content in feed and feces, the degradation of rutin in the gut was determined as follows:

$$\begin{array}{l} \;\mathrm{Degradation of rutin}\;(\%)=\\ \;\frac{\mathrm{rutin content in feed}\;(\mathrm{mg}/g)\times \mathrm{feed intake}\;(g)-}{\mathrm{rutin content in feed}\;(\mathrm{mg}/g)}\\ \frac{\mathrm{rutin contentin feces}\;(\mathrm{mg}/g)\times \mathrm{feces excretion}\;(g)}{\times \mathrm{feed intake}\;(g)}\times 100\% \end{array}$$

### Statistical Analysis

Data are expressed as means ± SEM. Data analysis was performed using SPSS 15.0 software (Chicago, IL, United States). Comparisons between groups were made using ANOVA followed by *post hoc* test and associations were assessed by the linear regression. Correlation and regression analysis of the microbiome and relative parameters were conducted using Spearman’s rank correlation coefficient. For the gene expression data analysis, the expression of each gene was normalized to the housekeeping gene β-actin (Ct_Target gene_-Ct_β–actin_). Statistical analyses were performed based on ΔCt. The relative abundance of relative gene expression was reported as 2^−ΔΔCt^, where ΔΔCt = ΔCt_Experiment_-ΔCt_Control_.

## Results

### Food Consumption and Animal Growth

**FIGURE 1:**
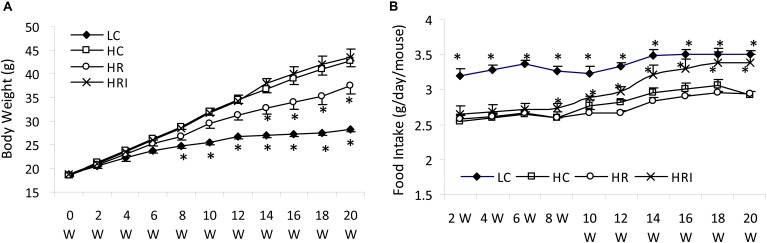
Effect of high fat diet, rutin and inulin supplementation on body weight **(A)** and food intake **(B)**. Rutin supplement ameliorated the elevation of body weight induced by high-fat diet, but its co-administration with inulin elevated gradually food intake toward no significant effect on body weight compared with HC. Black star indicated there was a significant difference compared with HC (*p* < 0.05).

The body weights between the HC and HRI groups were similar (*p* > 0.05), but higher than that of LC group after 8 weeks and that of HR group after 14 weeks (*p <* 0.05, Figure 1A). As expected, the LC group had the highest food intake during the experiment and the HC group has the lowest (Figure 1B). The HR group had the similar food intake to the HC group (*p* > 0.05) whereas the further supplementation with inulin (the HRI group) increased food intake gradually, reaching to a significant level at 8 weeks when compared to the HC and HR group (*p <* 0.01), and close to LC after 16 weeks.

### Plasma Metabolic Parameters

High fat diet (the HC group) increased plasma cholesterol, triglycerides, leptin and LPS contents when compared to the LC group (Table 1). The rutin supplementation numerically reduced the plasma triglyceride (HR vs. HC), and its co-administration with inulin magnified this effect into a statistically significant level (*p <* 0.05, HRI vs. HC). Surprisingly, the cholesterol contents were not reduced by rutin (HR vs. HC) and even increased by inulin (HRI vs. HC). The leptin content was reduced by rutin supplementation and the LPS contents were significantly reduced in HR and HRI groups when compared to the HC group (*p <* 0.05).

**Table 1:** Effect of high fat diet, rutin, and inulin supplementation on plasma biochemical parameters.

	LC	HC	HR	HRI
Plasma Cholesterol (mmol/L)	4.45 ± 0.11^a^	6.48 ± 0.15^b^	6.33 ± 0.52^b^	7.63 ± 0.27^c^
Plasma TG (mmol/L)	0.73 ± 0.03^a^	1.19 ± 0.04^b^	1.06 ± 0.03^bc^	1.00 ± 0.02^c^
Plasma Leptin (ng/ml)	1.89 ± 0.24^a^	22.22 ± 2.10^b^	14.56 ± 2.83^c^	21.87 ± 2.04^b^
Plasma LPS (EU/L)	772.6 ± 32.8^a^	969.6 ± 36.3^b^	772.4 ± 54.8^a^	817.2 ± 52.3^a^

*LC, low-fat control group; HC, high-fat control group; HR, the group fed high-fat diet with rutin; HRI, the group fed high-fat diet with rutin and inulin. In each line, the values with different letters indicated significant difference among groups (p < 0.05).*

### The Inflammatory Factors in the Blood and Small Intestine

**FIGURE 2:**
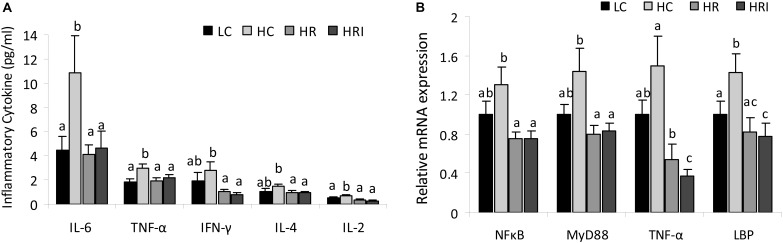
Effect of high fat diet, rutin and inulin supplementation on inflammatory cytokine profile. **(A)** Plasma inflammatory cytokine levels across the groups; **(B)** mRNA expression levels of inflammatory mediators in the small intestine tissue. The values with different letters indicated significant difference among groups (*p* < 0.05).

Five plasma inflammatory cytokines, IFN-γ, IL-4, IL-6, IL-2, and TNF-α, were measured using the ProcartaPlex Multiplex Immunoassay (Figure 2A). Compared to the LC group, the high fat diet (the HC group) increased plasma levels of those cytokines, with statistically significant differences for IL-6, TNF-α and IL-2 (*p <* 0.05), and the supplementation with rutin or rutin + inulin significantly reduced the plasma concentrations of those cytokines (HR and HRI vs. HC, *p <* 0.01). For the inflammatory mediators (Figure 2B), NFκB, MyD 88, TNF-α and LBP, the mRNA levels of them were higher in the HC group when compared to the LC group with a statistical difference for LBP, the gene for LPS binding protein (*p <* 0.05). The rutin supplement or its combination with inulin suppressed the production of these mediators (HR and HRI vs. HC, *p <* 0.05), and notably the combinatorial supplementation of rutin and inulin significantly reduced the intestinal expression of TNF-α (HRI vs. HR, *p <* 0.05).

### Expression of Paneth Cell AMPs and ER Stress Markers

**FIGURE 3:**
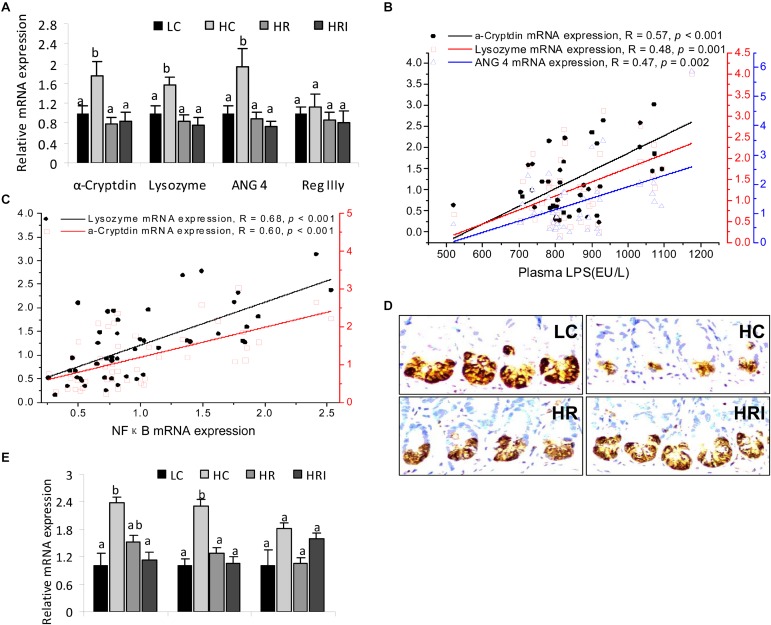
Effect of high fat diet, rutin, and inulin supplementation on the expression of Paneth cell antimicrobial peptides (AMPs) and ER stress biomarkers in intestine tissue. **(A)** mRNA expression of Paneth cell AMPs; **(B)** the correlations of Paneth cell AMPs with plasma LPS; **(C)** the correlations of Paneth cell AMPs with mRNA expressions of NFκB; **(D)** Immunohistochemtry for lysozyme; **(E)** mRNA expression of ER stress biomarkers. The values with different letters indicated significant difference among groups (*p*<0.05).

The relative mRNA expression levels of Paneth cell antimicrobial peptides (AMPs) were detected in the small intestinal tissue (Figure 3A). Except for Reg IIIγ, high fat diet (the HC group) significantly increased mRNA expressions of Paneth AMPs: α-cryptdin (1.8-fold), lysozyme (1.6-fold), and ANG 4 (1.9-fold) (*p* < 0.05). The mRNA expression levels of these Paneth cell AMPs were positively correlated with the plasma LPS level (Figure 3B, *p <* 0.01) and the gene expressions of inflammatory mediators NFκB (Figure 3C, *p <* 0.01).

However, when we measured the protein level of lysozyme by immunohistochemistry (Figure 3D) the results indicated HC had lower protein expression in Paneth cells when compared to the LC group, indicating discordance between protein expression and mRNA expression of lysozyme in the Paneth cells. Since endoplasmic reticulum directly participates in the translational expression of those AMPs and may be responsible for the inconsistency at the protein and mRNA level, we therefore detected mRNA expression of ER stress markers in the intestine tissue, such as chaperone protein binding protein (BiP), activating transcription factor 4 (ATF4) and c/EBP-homologous protein (CHOP). The results showed higher mRNA expressions of BiP and ATF4 in HC when compared to the LC group (Figure 3E).

With the addition of rutin or its combination with inulin ameliorated the elevation of the mRNA expression of Paneth AMPs and ER stress biomarkers, and reduction of the lysozyme protein expression induced high-fat diet (Figures 3A,D,E).

### The Abundances of Gut Microflora

**FIGURE 4:**
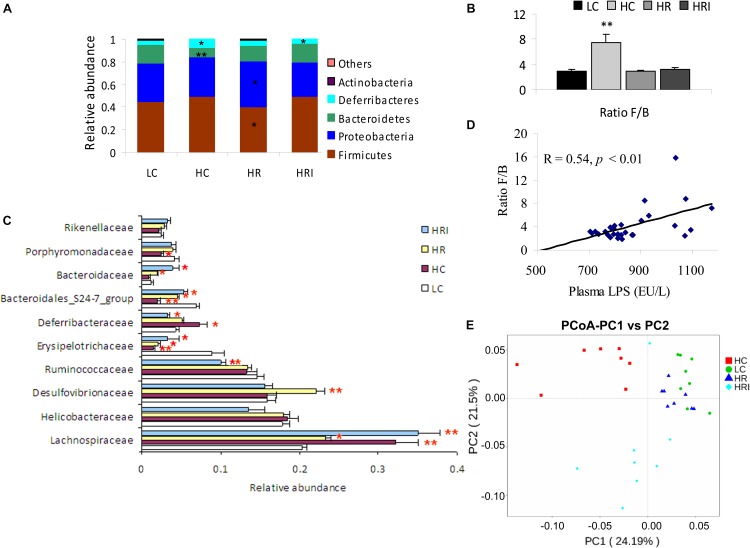
Effect of high fat diet, rutin and inulin supplementation on the composition of gut microflora. **(A)** Taxonomic composition at the phylum level, the abundance ratio of each phylum was based on total microorganisms; **(B)** *Firmicutes*/*Bacteroidetes* (F/B) ratio; **(C)** The relative abundance of top 10 families; **(D)** Ratio F/B was positively related with plasma LPS; **(E)** Principal coordinate analysis (PCoA) of 16S sequences from 32 intestine content samples of four treatments based on Weighted Unifrac. The microbiome of HR was similar to LC, HF feeding reduced significantly the abundance of *Bacteroidetes* and its families, favoring the presence of *Lachnospiraceae* family in *Firmicutes* and *Deferribacteres*, and resulted in higher *Firmicutes*/*Bacteroidetes* (F/B) ratio in intestine, while HR and HRI group increased the abundance of *Bacteroidetes* and its families, and attenuated the rise of F/B ratio and *Deferribacteres*, and HR group increased the growth of *Proteobacteria* and its family. ^∗^ indicated significant difference at *p* < 0.05, and ^∗∗^ indicated significant difference at *p* < 0.01 compared with LC.

**FIGURE 5:**
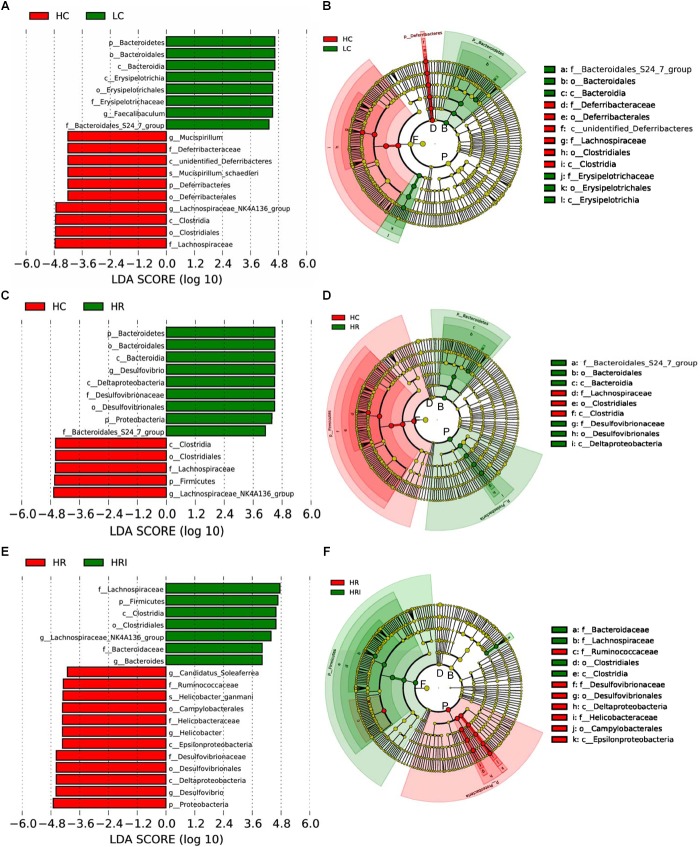
Linear discriminant analysis (LDA) effect size (LEfSe) analyses compared the alterations in microbiome according to the diet. Output showing effect size of significantly enriched taxa when comparing groups: HC vs. LC **(A)**, HC vs. HR **(B)** and HR vs. HRI **(C)**. Significant taxa plotted onto a cladogram when comparing groups: HC vs. LC **(D)**, HC vs. HR **(E)**, and HR vs. HRI **(F)**. F, *Firmicutes*; P, *Proteobacteria*; B, *Bacteroidetes*; D, *Deferribacteres;* p_, phylum; c_, class; o_, order; f_, family; g_genus, s_, species.

High fat diet (the HC group) significantly reduced the *Bacteroidetes* (*p* < 0.01)and its families (especially *Bacteroidales_* S24-7 group, *p* < 0.001*, Porphyromonadaceae*, *p* < 0.05), in favor of the presence of *Lachnospiraceae* family in *Firmicutes* (*p* < 0.01) and *Deferribacteres* (largely due to increase of *Deferribacteraceae* family, *p* < 0.05), and resulted in an increased *Firmicutes*/*Bacteroidetes* (F/B) ratio (*p* < 0.01) (Figures 4A–C, 5A,B). However, high fat diet reduced *Erysipelotrichaceae* in *Firmicutes* phylum (*p* < 0.01) in the intestine when comparing to the LC group (Figure 4C). Correlation analysis indicated F/B ratio was positively associated with plasma LPS level (Figure 4D, *p* < 0.01). Rutin supplementation or its combinational supplement with inulin promoted the growth of the most families of *Bacteroidetes* (Figures 4A,C), such as *Bacteroidales_*S24-7 group (HR and HRI vs. HC, *p <* 0.001), *Bacteroidaceae* (HR and HRI vs. HC, *p <* 0.01), *Porphyromonadaceae* (HR vs. HC, *p* < 0.01; HRI vs. HC, *p* = 0.09), and *Rikenellaceae* (HR vs. HC, *p* < 0.05; HRI vs. HC, *p* = 0.05), and decreased the F/B ratio (Figure 4B, HR and HRI vs. HC, *p <* 0.01) and *Deferribacteraceae* population (Figures 4A,C, HR vs. HC, *p* = 0.05; HRI vs. HC, *p* < 0.01). Rutin supplementation alone (the HR group) reduced the number of *Firmicutes* (*p <* 0.01), especially *Lachnospiraceae* family (*p <* 0.05), and promoted the growth of *Proteobacteria* phylum (*p <* 0.05) (largely due to *Desulfovibrionaceae* family) in comparison with HC group (Figures 4A,C, 5C,D). The further supplementation with inulin (the HRI group) altered the composition of gut microflora (Figures 4C, 5E,F): increased the proportions of *Lachnospiraceae* (*p* < 0.01) and *Bacteroidaceae* (*p* < 0.05), and suppressed *Desulfovibrionaceae* (*p* < 0.001), *Ruminococcaceae* (*p* < 0.01), and *Deferribacteracea*e (*p* < 0.001) at family level; at genus level, elevated (*p* < 0.05) the abundances of *Lachnospiraceae*_NK4A136_group, *Lachnoclostridium*, *Roseburia*, *Blautia*, *Bacteroides* and *Lactobacillus*, and reduced (*p* < 0.01) *Desulfovibrio* *Ruminiclostridium*_9 and *Mucispirillum* (Supplementary Figure S1) when compared with HR group.

The characteristics of the gut microbiota in LC group and rutin-supplemented mice were similar according to PCoA plot (Figure 4E), besides rutin supplementation increased significantly *Proteobacteria* phylum (Figure 4A), and they were far away from HC and HRI groups.

### The Content of SCFA and Rutin in Feces

High fat diet feeding (the HC group) reduced acetate, propionate, butyrate and their total contents in colonic contents when compared to the LC control group (Table 2). The supplementation of rutin or its combination with inulin significantly attenuated the reduction of SCFA. When compared to the HC group, the rutin supplementation (HR group) increased acetate and propionate contents (*p* < 0.05), and its co-administration with inulin (the HRI group) increased the production of all 3 SCFAs (*p <* 0.05). It is highly noteworthy that the co-administration with inulin (the HRI group) further significantly increased the production of butyrate (*p <* 0.05), which is considered as the key SCFA, and numerically increased propionate (*p* = 0.10) when compared to the HR group.

**Table 2:** Effect of high fat diet, rutin and inulin supplementation on colonic contents of SCFA in mice (μmol/g).

	LC	HC	HR	HRI
Acetate	9.52 ± 0.88^a^	7.04 ± 0.48^b^	9.23 ± 0.99^a^	9.20 ± 0.78^a^
Propionate	1.43 ± 0.23^a^	0.95 ± 0.08^b^	1.53 ± 0.15^a^	1.92 ± 0.19^a^
Butyrate	2.75 ± 0.79^a^	1.12 ± 0.05^b^	1.28 ± 0.17^b^	1.79 ± 0.20^c^
Total	13.69 ± 1.23^a^	9.10 ± 0.45^b^	12.04 ± 1.12^a^	12.91 ± 1.06^a^

*In each line, the values with different letters indicated significant difference among groups (p<0.05).*

The HRI group indicated lower rutin left in feces (2.16 ± 0.47 vs. 2.90 ± 0.09 mg/g, *p* < 0.05), and increased the catabolism (the % of degradation) of rutin (95.37 ± 0.79 vs. 92.42 ± 0.64%, *p* < 0.05) in the feces compared with HR group.

## Discussion

The dysbiosis of gut microbiota is an important mediator in the high fat-induced inflammation, and thus manipulation of the gut microbiota, using prebiotics, may provide novel preventive strategies for obesity-associated inflammation and medical disorders. Both polyphenols and fibers, often eaten together, have been reported to have prebiotic actions. The present study demonstrated that the supplementation of polyphenol rutin suppressed the rise of F/B ratio, *Deferribacteracea* population and plasma LPS induced by HF diet, and thereby mitigated systematic and intestinal inflammation, and ER stress in Paneth cells. The co-administration with polysaccharide inulin improved the utilization of rutin and further increased the production of butyrate and reduced the expression of the key inflammatory cytokine TNF-α, indicating the polyphenol rutin and polysaccharide inulin can be utilized combinatorially as a dietary strategy to ameliorate gut dysbiosis and inflammation associated with HF-induced obesity.

In the present study, after feeding a 60 kcal% HF diet for 20 weeks, the animals exhibited a significant increase of body weight with increased plasma cholesterol, triglycerides, leptin and LPS contents, and displayed a systematic and intestinal inflammation as indicated by increased circulating inflammatory cytokines and elevated expressions of inflammatory mediators in the intestinal epithelial cells. Meanwhile, HF diet significantly increased mRNA expressions of Paneth AMPs, which were positively associated with plasma LPS and inflammatory mediators, suggesting that a critical role of Paneth cell AMPs in promoting obesity-associated inflammation. HF diet also induced a significantly increase of ER stress as indicated by the higher mRNA expressions of ER stress biomarkers, BiP and ATF4, which resulted in a discordance between protein expression and mRNA expression of lysozyme. Similarly, Hodin et al. (2011) reported ER stress was apparent in Paneth cells in obese subjects, indicated by diminished AMPs protein expression and increased mRNA levels of their corresponding genes.

Corresponding to those metabolic and inflammatory changes, a shift of the gut microbiota was observed in the animals fed with HF diet comparing to the LF diet group, with elevated F/B ratio, similar to the alteration observed in obese individuals induced by high-fat (Zhang et al., 2012; Guo et al., 2017) and high fat/high-sucrose diet (Parks et al., 2013). The increase of *Firmicutes* in HC was largely due to increase of *Lachnospiraceae* in this study, which was similar to results of Han et al. (2018) indicating *Lachnospiraceae* is the main intestinal bacterial family, and accounts over thirty percent of the total in the microbiota. Correlation analysis indicated F/B ratio and abundance of *Lachnospiraceae* (*R* = 0.41, *p* < 0.05) were correlated with plasma LPS in the present study. These findings were consistent with previous studies, which demonstrated that HF diet-induced gut microbiota dysbiosis (shifting to *Firmicutes*) led to an increase in gut permeability and plasma LPS concentration, and thereby promoted a low-grade inflammation (Cani et al., 2007, 2008; Creely et al., 2007).

Rutin supplementation (HR group) or its co-administration with inulin (HRI group) mitigated the increase of plasma triglycerides or leptin, attenuated inflammatory status, and improved the ER stress in Paneth cells induced by high-fat diet (the HC group) in this study. These data suggested that the inflammatory status and ER stress in Paneth cells in an obese state could be compromised by rutin supplementation or its co-administration with inulin in HF diet.

The characteristics of the gut microbiota in rutin-supplemented mice were similar to LC group according to PCoA plot, suggesting the addition of rutin could attenuate gut dysbiosis induced by HF diet. Rutin supplementation in HF diet suppressed the reduction of *Bacteroidetes* and its families, and ameliorated the elevation of *Lachnospiraceae*, *Firmicutes*/*Bacteroidetes* (F/B) ratio, *Deferribacteraceae* and plasma LPS induced by high-fat diet. Our finding was supported by a number of previous studies that demonstrated that the influences of polyphenols on intestinal bacteria: purple lettuces administration, which contains high flavonoid content, decreased *Lachnospiraceae* and *Deferribacteraceae* (Han et al., 2018)*;* regular wine vinegar ingestion or polyphenol-rich fruits and green tea favored the growth of *Bacteroidetes* community (Lee et al., 2006; Cerezo et al., 2008; Selma et al., 2009); and supplementation of quercetin or grape polyphenols stimulated the proliferation of *bifidobacteria* and attenuated the rise of F/B ratio and thereby ameliorated obesity-associated inflammation and metabolic disorders (Parkar et al., 2013; Etxeberria et al., 2015; Roopchand et al., 2015).

Polyphenols and dietary fiber are often eaten together, and interact to mediate gut microbiota. In the present study, the co-administration of polysaccharide inulin with rutin increased the breakdown of the rutin, indicated by the lower rutin left in feces when comparing the HRI with the HR group. This result was consistent with a prior *in vitro* study, which demonstrated that fermentable fibers could speed up the breakdown of the rutin (Jaganath et al., 2006). As expected, the combinatorial supplementation of inulin with rutin magnified the effects of rutin supplementation alone: elevated the production of butyrate and reduced the intestinal expression of TNF-α (*p* < 0.05).

Unfavorably, in this study, rutin supplement ameliorated the increase of body weight triggered by high-fat diet, while the co-administration with inulin reversed the decrease of body weight induced by rutin supplementation. This might largely be due to food intake elevated by sweet taste of inulin. Nevertheless, the combination of rutin and inulin further elevated the growth of *Bacteroidaceae* and *Lactobacillus*, and recovered the numbers of SCFA -producing bacterium *Lachnospiraceae* reduced by rutin addition (Figure 4C and Supplementary Figure S1). The bloom of *Lachnospiraceae* was largely accounted by increases of *Lachnospiraceae*_NK4A136_group, *Lachnoclostridium*, *Roseburia* and *Blautia*, most of which are butyrate producers (Million et al., 2018). Previous studies also demonstrated that inulin supplementation favored *Lactobacillus* and SCFA-producing bacteria *Lachnospiraceae* or *Roseburia*, and *Bacteroides* (Aguirre et al., 2016; Zhang et al., 2018). The HRI group also reduced a number of harmful bacteria including *Deferribacteraceae* and *Desulfovibrionaceae.* The further decrease in *Deferribacteraceae* in HRI was largely accounted for reduction of *Mucispirillum* genus in this study, which is known as mucin degrader and associated with early disruption of the colonic surface mucus layer, prior to the onset of symptomatic colitis (Belzer et al., 2014). Most members of *Desulfovibrionaceae* are reported as lipopolysaccharide (LPS) producers and lead to a low grade and chronic inflammation in obese objects (Xiao et al., 2014; Zhang-Sun et al., 2015). These results were in agreement with previous studies that reported *Mucispirillum* (belong to *Deferribacteracea)* and *Desulfovibrionaceae* abundances were significantly suppressed by inulin treatment (Yu et al., 2018; Zhang et al., 2018).

## Conclusion

In summary, the present study demonstrated that rutin supplementation suppressed the rise of F/B ratio, *Deferribacteracea* and plasma LPS induced by HF diet, and thereby mitigated systematic and intestinal inflammation, and ER stress in Paneth cells. The co-administration of rutin with inulin improved the utilization of rutin as indicated by its decreased excretion, further suppressed a number of harmful bacteria, reduced the expression of the key inflammatory cytokine TNF-α and increased the production of butyrate. Taken together, our data demonstrated the potential to combine polyphenol rutin and the polysaccharide inulin as a dietary strategy to ameliorate gut dysbiosis, to improve inflammatory status and thereby to reduce medical disorders associated with HF-induced obesity.

## Ethics Statement

This study was carried out in accordance with the laboratory animal-guildline for ethical review for animal welfare in China. The animal protocol was approved by the Institutional Animal Care and Use Committee of Chengdu University (Permission No. 2018-23-01).

## Author Contributions

XG and ZL designed the research. YL and JL performed the research. RT and SY analyzed the data. XG wrote the paper. ZL supervised the manuscript.

## Conflict of Interest Statement

The authors declare that the research was conducted in the absence of any commercial or financial relationships that could be construed as a potential conflict of interest. The handling Editor declared a shared affiliation, though no other collaboration, with one of the authors SY at the time of review.

## Footnotes

1 http://huttenhower.sph.harvard.edu/galaxy/
