# Supplementary figures and images for: Rutin and Its Combination With Inulin Attenuate Gut Dysbiosis, the Inflammatory Status and Endoplasmic Reticulum Stress in Paneth Cells of Obese Mice Induced by High-Fat Diet
Source: Front Microbiol. 2018 Nov 5;9:2651. doi: 10.3389/fmicb.2018.02651 (PMC6230659; doi:10.3389/fmicb.2018.02651)

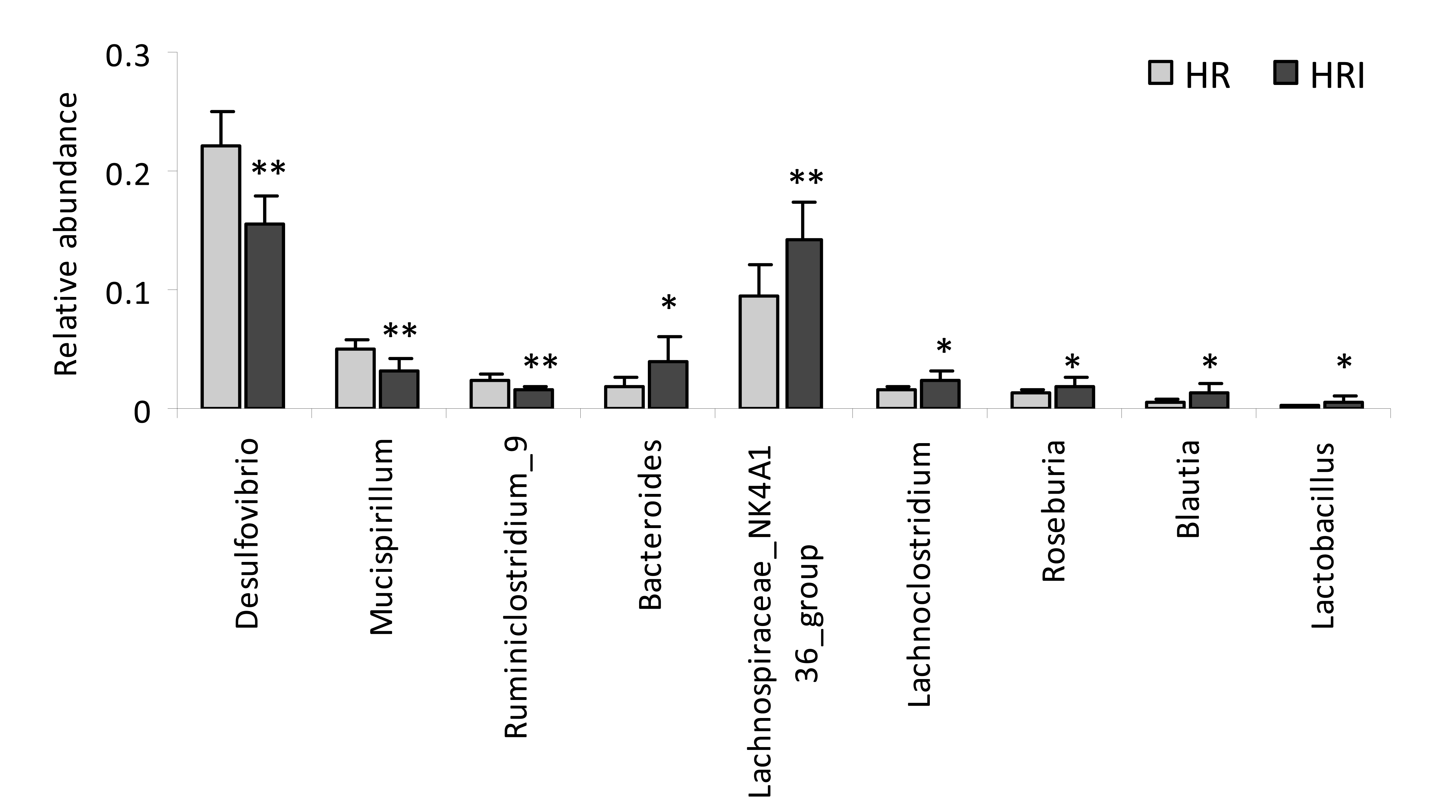

Supplement: Supplementary file 1 [file Image_1.TIF]
