# Supplementary material for: Rutin and Its Combination With Inulin Attenuate Gut Dysbiosis, the Inflammatory Status and Endoplasmic Reticulum Stress in Paneth Cells of Obese Mice Induced by High-Fat Diet
Source: Front Microbiol. 2018 Nov 5;9:2651. doi: 10.3389/fmicb.2018.02651 (PMC6230659; doi:10.3389/fmicb.2018.02651)
Supplement: Supplementary file 2 [file Table_1.DOCX]

**Supplementary Data_Table S1:** Primer sequences used for the quantification of the expressions of inflammatory factor, antimicrobial peptides and ER stress markers in small intestinal mucosa by real-time quantitative PCR.

| **Genes** | **Forward Primer** | **Reverse Primer** |
| --- | --- | --- |
| TNF-α | GACAAGGCTGCCCCGACTA | CGGCAGAGAGGAGGTTGACT |
| NFκβ | TGAGCCCTTCCTGGATTTCC | CCTAGGTCATTCTGCAGGTCAAG |
| MyD88 | AGAGCTGCTGGCCTTGTTAG | TTCTCGGACTCCTGGTTCTG |
| LBP | CTTGGCGTGGTCACTAATGTATTT | TGTGCCTTGTCTGGATGCA |
| Lysozyme | AGATCCCCAAGGCATTCGA | TCCTACAGTGAGAAAGAGACMGAAT |
| α-Cryptdin | CAGCCAGGAGAAGAGGACCAG | TAGCATACCAGATCTCTCAACGAT |
| Angiogenin 4 | TGTCCTTTGTTGTTGGTCTTCGT | GTGCTGACGTAGGAATTTTTCGT |
| RegIII γ | CCTTCCTGTCCTCCATGATCA | CCACTCCCATCCACCTCTGT |
| BiP | TCATCGGACGCACTTGGAA | TGGTTTGCCCACCTCCAATA |
| ATF4 | GGAAGACACTCCCTCTGACAATG | ACGGGAACCACCTGGAGAAG |
| CHOP | GCGGAAAGTGGCACAGCTA | TGATGCCCACTGTTCATGCT |
| β-Actin | GGCTGTATTCCCCTCCATCG | CCAGTTGGTAACAATGCCATGT |

**Figure S1** Effect of rutin supplementation alone or in combination with inulin on the composition of gut microflora at the genus level. * indicated significant difference at *p* < 0.05, and ** indicated significant difference at *p* < 0.01 compared with HR.
